# Synergies of Antiangiogenic Therapy and Immune Checkpoint Blockade in Renal Cell Carcinoma: From Theoretical Background to Clinical Reality

**DOI:** 10.3389/fonc.2020.01321

**Published:** 2020-07-29

**Authors:** Xiaohan Zhou, Wanting Hou, Ling Gao, Lin Shui, Cheng Yi, Hong Zhu

**Affiliations:** Department of Medical Oncology, Cancer Center, West China Hospital, Sichuan University, Chengdu, China

**Keywords:** metastatic renal cell carcinoma, tumor microenvironment, vascular endothelial growth factor (VEGF), antiangiogenic agents, immunotherapy, immune checkpoint inhibitors (ICI), immunomodulation

## Abstract

The hallmarks of renal cell carcinoma (RCC) are angiogenesis and immunogenic tumor microenvironment. Over the past decades, treatment options for metastatic RCC (mRCC) have been expanding, from the inhibition of vessel formation via antiangiogenic agents (AAs) to the stimulation of immune system by immune checkpoint inhibitors (ICIs). Since 2005, the introduction of antiangiogenic agents targeting vascular endothelial growth factor (VEGF), its receptors (VEGFRs), and mammalian target of rapamycin (mTOR) pathway have experienced moderate success in the therapeutics of mRCC, but patient outcomes remain suboptimal. Recently, the development of ICIs targeting cytotoxic T-lymphocyte-associated protein 4 (CTLA-4) and the programmed death-1/programmed death ligand 1 (PD-1/PD-L1) pathways has dramatically changed the treatment landscape of mRCC. Expressly, the combination of ipilimumab and nivolumab has been confirmed to improve clinical outcomes and approved as a standard care for intermediate- or poor-risk mRCC patients. Nevertheless, innate or adaptive drug resistance is observed within both treatment approaches, limiting overall clinical benefit. This phenomenon will underscore the urgent need for new combinational therapy strategies with different mechanisms of action, which can improve efficacy in an extended patient population without severe toxic effects. In 2019, as the results of two critical phase III trials came to light, FDA approved axitinib plus avelumab, or pembrolizumab as first-line standard management for mRCC, which cements the combination of AAs plus ICIs and advances the mRCC treatment field. This review summarizes current evidence on the interplay and synergies between AAs and immunomodulating drugs in mRCC, focusing on the theoretical background and the status of current clinical development.

## Introduction

Kidney cancer is amid the ten most common cancers in both men and women, with more than 400,000 cases worldwide in 2018 (1). RCC is the predominant type of kidney cancer, and 85% of RCC is identified as the ccRCC histological subtype (2). In recent years, it was observed that incidence rates of RCC have been climbing. Part of the reason is the incidental detection of renal masses during abdominal imaging among higher-income settings. Though most detected tumors are small, the locally advanced disease was still diagnosed in a striking proportion of patients, with up to 17% of patients initially diagnosed with distant metastases (3). RCC remains a poor prognosis, despite the progress in RCC diagnosis and management over the past two decades. Locally advanced and mRCC have historically been challenging to treat, due to characteristically resistance to standard chemotherapies, radiotherapies, and hormonal therapies, with treatment options traditionally limited (4). Before the development of targeted therapy, cytokine-based (IL-2 and IFN-α) immunotherapy was approved as the standard care for mRCC, despite the durable long-term responses and prolonged survival in a tiny proportion of patients. These agents bring about significant toxicity by activating the immune system in a non-specific manner, and are unable to identify which patients are likely to respond. Thus, the future widespread use has been precluded (5–7).

As our basic knowledge on RCC tumorigenesis increased, it has translated into the initially successful development and application of targeted agents in RCC management. In particular, VEGF-targeting antiangiogenic agents have been most widely used in the first-line treatment setting. Sorafenib gained FDA approval in 2005, quickly followed by others, including sunitinib, pazopanib, axitinib, and cabozantinib (8–12). Though antiangiogenic therapy can provide remarkable short-term clinical benefits in objective response rate (ORR) and progression-free survival (PFS), it commonly develops into therapeutic resistance, and hence rarely produce enduring long-term responses or survivals (13).

**Figure 1:**
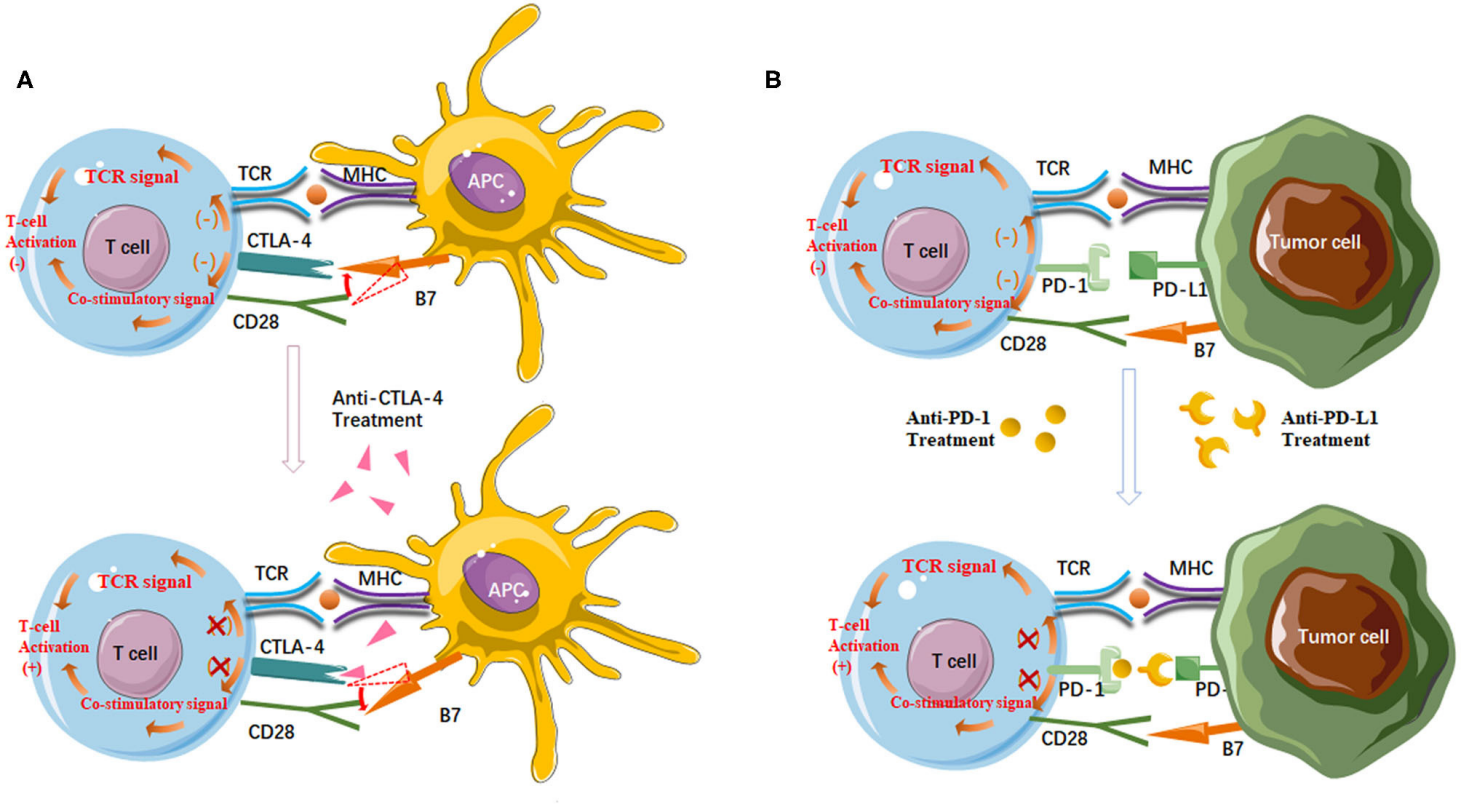
**(A)** Anti-CTLA-4 agents enhance T cell activation. CTLA-4 has a higher affinity for B7 than CD28. CTLA-4/B7 ligation reduces CD28/B7 ligation and then suppresses T cell activation. On the other hand, the interaction between CTLA-4 and B7 inhibits the stimulatory signals produced by TCR/MHC and CD28/B7 binding. Antibodies targeting CTLA-4 deplete CTLA-4 from the tumor microenvironment and thereby enhance T cell activation; **(B)** Anti-PD-1/PD-L1 agents enhance the anticancer activity of T cells. PD-1/PD-L1 ligation inhibits T cell activation and TCR signal. Antibodies targeting PD-1 or PD-L1 can block the PD-1/PD-L1 pathway to reactivate tumor antigen recognition as well as the proliferation, infiltration, and activation of cytotoxic CD8^+^ T cells.

Based on a deeper understanding of T cell activation, the establishment of ICI has brought a golden era in mRCC therapeutic landscape (14, 15). The two main types of ICIs target CTLA-4 and PD-1/PD-L1 (Figure 1). CTLA-4 is a receptor present on T cells where it acts as a brake on T cell activation. Thus, inhibition of CTLA-4 aims at a relief of this kind of obstacle, and allowing the native immune system to instigate antitumor immunity. PD-1 is also present on T cells, leading to down-regulation of immune responses when bound to its ligand PD-L1. Tumor cells often express PD-L1 to mediate this blockage. Thus, the strategy of inhibiting PD-1 or PD-L1 is to prevent this down-regulation of antitumor immunity (16). Nivolumab, a monoclonal antibody targeting PD-1, was the first ICI approved in mRCC treatment, showing longer overall survival (OS) of patients following antiangiogenic therapy, and fewer grade 3 or 4 adverse events occurred compared to everolimus (17, 18). Accumulating evidence, however, demonstrates that not all patients may benefit from single-agent immunotherapy, underscoring that combination regimens can improve efficacy in broader groups of patients without exacerbating toxic effects (19). Since then, multiple kinds of research that explore various ICI combination regimens have emerged (20).

The combination of nivolumab and ipilimumab was the first step in this direction. In CheckMate 214 (21), a pivotal phase III trial, a combination strategy, nivolumab plus ipilimumab, indicated statistically superior median OS and higher ORR in patients with intermediate- and poor-risk disease by international metastatic database consortium (IMDC) criteria compared with sunitinib, thus leading to the FDA approval as a first-line treatment in 2018.

Current clinical investigations have focused on evaluating combination regimens containing ICIs and VEGFR-directed tyrosine kinase inhibitors (TKIs). Last year, three crucial phase III trials released their preliminary result, and two of them shortly got FDA-approval as standard treatment approaches for mRCC in the first-line setting.

In this review, we discussed the appropriate use of combination therapy (AAs plus ICIs) in mRCC, and purveyed an overview of the mechanisms and preclinical rationale of this strategy, including the potential role that AAs may play on tumor microenvironment and host immunity. The progresses of clinical trials, efficacy evidence and current controversies of combination regimens were summarized and discussed also.

## Angiogenesis and Immune Suppression in RCC Microenvironment

ccRCC, the dominant histologic subtype of RCC, is closely associated with mutation or inactivation of the *von Hippel-Lindau* (*VHL*), tumor suppressor gene that encodes VHL protein which is a key component of the cellular oxygen-sensing pathway (22, 23). Specifically, VHL protein is a crucial member of the ubiquitin ligase complex which can degrade hypoxia-inducible factor (HIF) subunits HIF-1α. When *VHL* gene is lost or inactivated, HIF-1α over accumulates. Subsequently, HIF-1α over-accumulation drives the cellular hypoxic response, resulting in transcription of several target genes involved in angiogenesis, oxygen transport, glucose uptake and metabolism, cell proliferation, and chemotaxis, which leads to carcinogenesis eventually (24–26). Enhanced angiogenesis, therefore, is one of the signatures of clear-cell RCC. Meanwhile, this disbalance of pro- and anti-angiogenic factors brings about numerous structural and functional abnormalities in tumor vessels characterized by irregular shape, tortuousness, hyperpermeability, lack of pericytes. All these changes induce an abnormal blood flow with resultant tumor cell extravasation, T cell intratumoral infiltration, and altered antitumor agent delivery (27).

On the other hand, it is widely regarded that RCC has a unique immune microenvironment (28). Perhaps more than any other solid tumor type, RCC is infiltrated with immune cells of various phenotype and function (29, 30). Mass cytometry has been used to comprehensively described tumor resident T cell from 73 ccRCC patients with different stages. T cells were the most common immune subset (50%) followed by tumor-associated macrophages (25%), natural killer cells (9%), B cells (4%) and other subsets (31). In line with other evidence, tumor-infiltrating T cells were predominantly CD8^+^ cells. However, the high levels of CD8^+^ T cell infiltration in RCC is associated with worse outcome, which is in contrast to most other tumor types, indicating that infiltrating CD8^+^ T cell pool is probably dominated by nearly exhausted T cells (32, 33). This negative correlation is likely attributed to the high expression of PD-1, CTLA-4, other immune checkpoint proteins on these invading T cells, and the impaired cytolytic function through interaction with other cells in tumor environment, such as myeloid-derived suppressor cells (MDSCs), which proved to suppress T cell and dendritic cell (DC) function (34, 35). Indeed, many studies on RCC microenvironment have reported defective T cell with dysfunctional cytotoxicity and ineffective tumor cell killing (36–39).

Taken together, the TME of RCC features high levels of angiogenic mediators, chemokines, and incapacitated T cells affected by checkpoint regulation or the immunosuppressive effects of MDSCs.

## Biological Rationale for Combined AA and Immune-Activating Therapy

**Figure 2:**
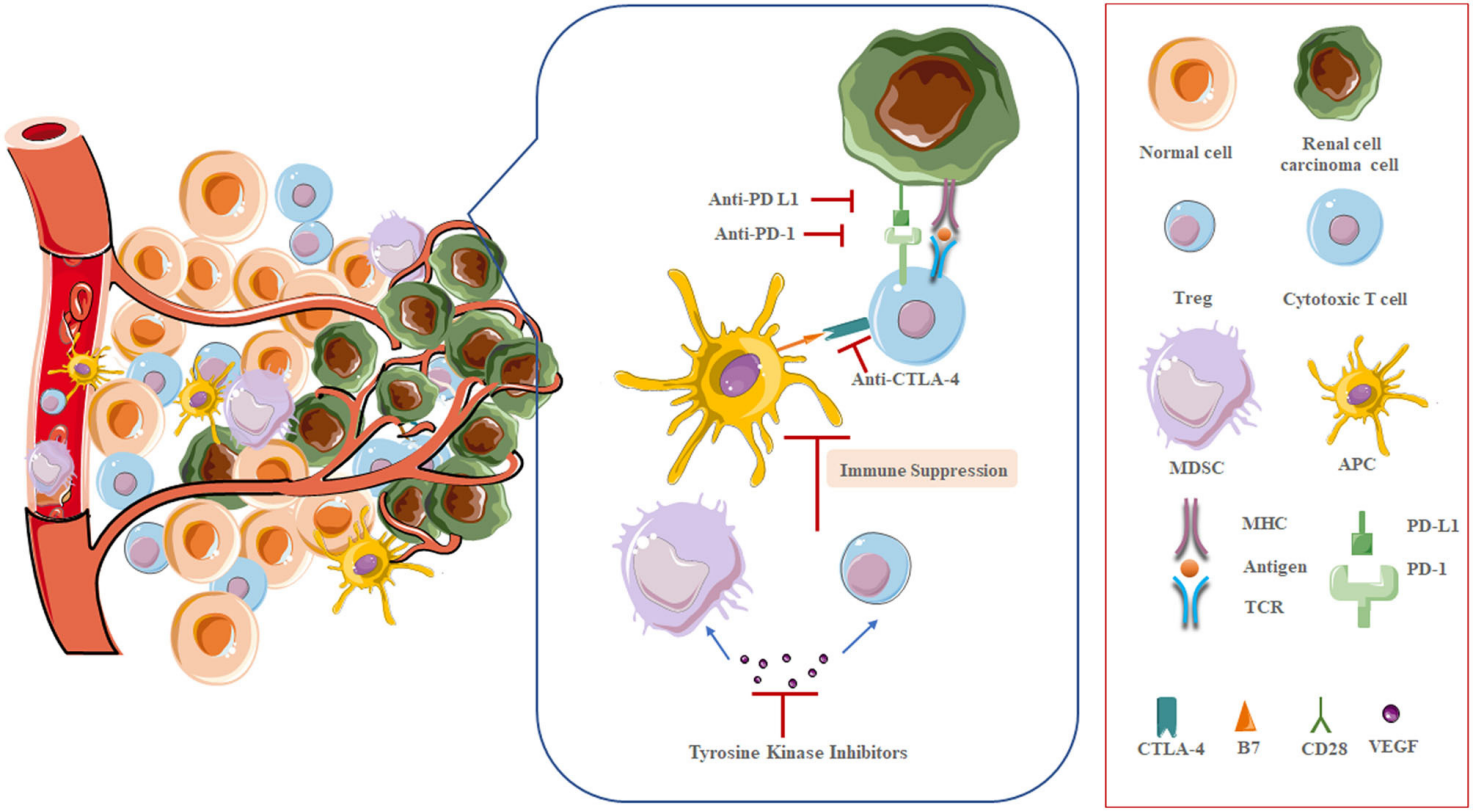
The interplay between the immune system and angiogenesis in renal cell carcinoma.

There is a broad sense that tumor immune evasion closely relates to angiogenesis, and, in turn, tumor angiogenesis highly depends on immunosuppressive microenvironment, which is companion processes in tumorigenesis (Figure 2) (40). Mounting evidence now show that elevated level of VEGF in tumor lesion may cause suppression in both innate and adaptive immune response, and increased serum or tumor VEGF levels are associated with unfavorable prognosis in mRCC patients (41). Reportedly, VEGF can inhibit the innate immune system via hampering the transcriptional program required for maturation of DCs, the critical cells in immune activation, and increasing the presence of MDSCs, which represent a heterogeneous population of cells that accumulate in tumor-bearing hosts, characteristic by their potent immune-suppressive activity against cytotoxic tumor-infiltrating lymphocytes (TIL) (42–45). VEGF also inhibits the adaptive immune system through blocking the differentiation of progenitor cells into CD4^+^ and CD8^+^T cells (46). Additionally, potent immunosuppressive factors, like inhibitory molecules on T cells (PD-1) and immunosuppressive cytokines (IL-10, TGF β), are also boosted by HIF-1α activation (47).

Multiple compelling data with promising insights into the potentiality of AAs, especially targeting VEGFR, as immunomodulators have been unveiled. Preliminary evidence has indeed demonstrated that anti-angiogenesis, especially targeting VEGF, might reverse the immunosuppression in tumor microenvironment of RCC, potentially promoting the efficacy of subsequent immunotherapy (48, 49). Initially, in clinic, the synergy of AA, bevacizumab, plus immunotherapy (IFN-α), although not by checkpoint inhibitors, has been demonstrated by two large randomized phase III trials in previously untreated mRCC patients, with improved PFS and ORR compared with IFN-α alone, leading to FDA-approval as a first-line treatment option in mRCC (6, 50). Permanently discontinued treatment caused by treatment-related adverse events were frequently observed in combined treatment groups.

At present, the development of ICIs provides us a new chance to reconsider the balance between efficacy and toxicity.

A preclinical study in mice reported that tumor-associated VEGF enhanced expression of PD-1 and other inhibitory checkpoints involved in CD8^+^ T cell exhaustion, such as CTLA-4, TIM-3, and LAG-3, and targeting VEGF reverted expression of inhibitory checkpoints (51). Similarly, another preclinical mice RCC model showed synergistic antitumor activity of the combination of suntinib plus a murine anti-PD-1 antibody, with greater numbers of tumor infiltrating lymphocytes than any other controls treated with each agent alone (52). In 2008, for the first time it was shown in 42 mRCC patients, that sunitinib decreased the presence of regulatory T cells (Treg) and MDSCs in the peripheral blood, accompanied by an improved Th1 response and reverses type-1 immune suppression in mRCC patients (53). Consistently, in 2009, a study conducted by Ko et al. indicated that sunitinib-based treatment has the potential to regulate antitumor immunity by decreasing the absolute number and percentage of circulating Tregs and MDSCs, demonstrating reversal in immune suppression. In 23 mRCC patients receiving to 50 mg sunitinib daily for 28 days followed by 14 days of rest for 6 weeks (54). Research exploring microenvironmental immune components demonstrated potential synergism of VEGF-TKI and anti-PD-L1 treatment in a neoadjuvant setting (55). The expansion of CD8^+^ TIL and reduction in MDSCs were observed in tumor digests from RCC patients who received sunitinib prior to the surgery, compared with those who were treatment-naïve. These TIL products contained more PD-1 expressing TIL, while the Treg infiltration was not altered. These data provide a rationale for combining sunitinib with PD-1/PD-L1 blockade, either synchronously, or sequentially.

Insight into how different AAs may vary in their competence in regulating TME is still limited. Unlike sunitinib, the detrimental effects of sorafenib on immune-modulating have been reported. This TKI seems to interfere with the progress of maturation and antigen presentation of DCs by downregulating the expression of major histocompatibility complex (MHC) and costimulatory molecules, as well as declining production of immunostimulatory cytokines (56). On the other hand, it was reported that sorafenib could decrease the percentage of circulating and tumor-infiltrating Tregs (57, 58), making the immune condition to a more stimulatory setting.

Regarding pazopanib, it was reported that it could reduce the expression of VEGFR1 and VEGFR2 to offset the immunosuppressive effects of VEGF, which affect DC maturation, and restore property DC immune stimulation by downregulating the Erk/β-catenin pathway (59, 60).

A small-scale study presented that pazopanib, but not sunitinib, sharply improved the antigen-presenting function of DCs, together with DC-maturation markers HLA-DR, CD40, and CCR7 upregulated, in RCC patients (61). This phenomenon may lie in the diversity of immune regulation effect and a similar pattern of receptor recognition displaying different affinities for VEGFR among agents (62). Besides, pazopanib decreases the absolute number of MDSCs, Tregs and CD14^+^ monocytes and triggers CD8^+^ lymphocytes and T cell memory Th1 response (63). Conflicting data also exist, a multicenter phase II trial showed a clear association between presurgical pazopanib and declined CD8^+^ T cell infiltration and increased PD-L1 expression by biomarker analysis from sequential tissue samples (64).

In terms of bevacizumab, data released by Wallin et al. (65) demonstrated that enhanced trafficking of lymphocytes and high CD8^+^ T cell infiltration had been found after combination therapy of bevacizumab plus atezolizumab in mRCC patients. Concomitantly, a related increase was observed in intra-tumoral major histocompatibility complex-1 (MHC-I) protein expression, Th1 and T-effector markers, and chemokines, especially CX3CL1. More recently, in neoadjuvant treatment, presurgical bevacizumab has been noted to reduce CD68^+^ macrophages in ccRCC patients, preventing macrophages cell-mediated immune-suppression (66). Cabozantinib has also been observed to increase intratumoral CD8^+^ T cell infiltration, as well as decrease the number and activity of MDSCs infiltration in several preclinical models which designed to explore the effects of cabozantinib on the immune cells function and immune microenvironment (67, 68).

## Current Therapeutic Landscape

A series of clinical trials have been conducted to evaluate the antineoplastic effects of ICIs and AAs combinations. In this review, a systematic study collection was performed from Cochrane Library, ClinicalTrials, MEDLINE and PubMed databases up to April 10th, 2020. Search terms we used included “renal cell carcinoma,” “nivolumab,” “pembrolizumab,” “atezolizumab,” “avelumab,” “durvalumab,” “ipilimumab,” or “immune checkpoint inhibitor” with “sunitinib,” “axitinib,” “cabozantinib,” “apatinib,” “pazopanib,” “lenvatinib,” “sorafenib,” or “bevacizumab,” and related and expanded MeSH terms. Also, we expanded our search to recent reviews. Studies involving concurrent intervention of ICIs and AAs were eligible. The population of interest was patients histologically or cytologically confirmed RCC with clear cell component or sarcomatoid features. Trials were excluded when involving other cancer patients or combining other kind of intervention unless outcome could be isolated.

In 2019, results of two widely anticipated trials were unveiled in the same issue of the New England Journal of Medicine. KEYNOTE-426 study is an open-label phase III study designed to assess the efficacy and safety of pembrolizumab plus axitinib vs. sunitinib monotherapy in treatment-naïve patients with mRCC (69, 70). After a median follow-up period of 12.8 months, pembrolizumab plus axitinib seemed to be superior to sunitinib only in ORR (59 vs. 35%) and mPFS (15.1 vs. 11.1 months; HR = 0.69, 95% CI 0.57–0.84) regardless of the risk groups. The most promising data set was the OS: 89% of combination arm and 78.3% of sunitinib control arm were alive after 12-month follow-up (HR for death, 0.53; 95% CI 0.38–0.74; *p* < 0.0001). Toxicities were comparable between the two groups.

In a similarly designed randomized multicenter phase III trial (Javelin Renal 101), 886 enrolled treatment-naïve patients with advanced RCC were assigned to receive either avelumab plus axitinib or sunitinib monotherapy (*n* = 444) (71). The co-primary endpoints were PFS and OS in the PD-L1 positive (PD-L1^+^) patients, which occupied 63% of the randomly selected population. PFS analysis was in favor of the combination regimen for PD-L1+ patients (13.8 vs. 7.2 months; HR = 0.61, 95% CI 0.475–0.790) and the overall population (13.8 vs. 8.4 months; HR = 0.69, 95% CI 0.563–0.840). The confirmed ORR in avelumab plus axitinib group and sunitinib group was 55.2 vs. 25.5%, respectively, and, above all, the efficacy of combination arms was consistent across the IMDC risk groups. As of the study reported, OS analysis was still immature. Taken together, results from these trials promoted recent FDA approvals of pembrolizumab or avelumab plus axitinib in the first-line management of advanced RCC, cementing the ICIs plus AAs strategy and improving the therapeutic landscape of mRCC.

Another phase III IMmotion151 trial compared the combination of atezolizumab plus bevacizumab to sunitinib as first-line management for mRCC patients (72). In the subset of patients who were PD-L1^+^ interim analysis confirmed that combinational treatment group prolonged PFS (11.2 vs. 7.7 months, HR 0.74; 95% CI 0.57–0.96) and ORR (43 vs. 35%), amid whom complete response reached 9%. Meanwhile, patients received atezolizumab plus bevacizumab suffered fewer grade 3/4 adverse events compared to sunitinib only (40 vs. 54%, respectively). Matured OS data are pending, and longer-term follow-up is required to evaluate whether a survival benefit will finally emerge (12).

It is worth mentioning that the tolerability and safety of newer combination regimens should be carefully balanced to that of monotherapies. CheckMate 016 (73) was the first clinical trial to examine the tolerability and safety of combination immunotherapy in advanced RCC. This multicenter phase I study had 5 treatment arms, including the combination of nivolumab with either TKIs, sunitinib, or pazopanib. This combination did exhibit antitumor efficacy. The ORR was 54.5% in nivolumab plus sunitinib (N+S) group and 45% in nivolumab plus pazopanib (N+P) group, but hepatic and renal toxicity was much higher than expected. Reportedly, all patients assigned to the ICIs plus VEGF–TKIs combination arms experienced treatment-related adverse events, with 81.8 and 70% of patients developing grade 3–4 side events, in N+S and N+P arm, respectively. The most common grade 3–4 adverse events were hypertension, liver enzymes rise, hyponatremia, and lymphocytopenia. Nearly one-third of patients in both groups permanently discontinued treatment because of treatment-related adverse events, although combined therapy brought higher response rates compared to monotherapy. To our knowledge, only one study, a phase I dose-escalation trial, investigated antiangiogenic therapy (sunitinib) in combination with tremelimumab, a monoclonal antibody against CTLA-4. For nine of 21 patients evaluable for response, the ORR was 43%, and disease stabilization was 33%, but due to unexpected and surprising toxicity, like acute renal failure and death, this combination therapeutic strategy was not considered worthy of further exploration (74). Another phase I/II trial (Keynote-018, NCT02014636) examining the combination of pembrolizumab plus pazopanib released its results at the American Society of Clinical Oncology (ASCO) Conference in June 2017. Similarly, these results suggested that this kind of combination is unsafe and reported significant concerns with regard to hepatotoxicity, despite improved response rates (75).

Multiple ongoing and future trials are exploring the role of various drug combinations (Table 1). There are two other keenly anticipated phase III trials in the pipeline, CheckMate 9ER (NCT03141177) and KEYNOTE-581/CLEAR (NCT02811861). As of right now, neither of them has mature data available. CLEAR, a multicenter, open-label, phase III trial has three arms, exploring pembrolizumab plus lenvatinib, or everolimus plus lenvatinib, or sunitinib monotherapy in the first-line mRCC setting. Its preceding phase II study unveiled that combination of pembrolizumab and lenvatinib offered an improved median PFS of 17.7 months as well as an improved ORR of 66.7% with tolerable toxicity (76). The primary endpoint of this ongoing phase III trial will be PFS with secondary endpoints as OS, ORR, health-related quality of life (HRQoL), and safety profiles and the estimated completion date will be in 2022. CheckMate 9ER, a two-armed phase III randomized study, is assessing nivolumab plus cabozantinib vs. sunitinib monotherapy in 701 previously untreated mRCC patients (77). Revealingly, a phase I trial focusing on the same combination strategy released impressive antitumor efficacy with pretreated mRCC patients enrolled (78). The last ICI and AA combinational regimen brought together pembrolizumab and cabozantinib. Although available data are from the phase I setting, these results are promising with the chance to improve patient care in the future (79). Enrollment of a phase II dose expansion is now ongoing (NCT03149822).

**Table 1:** Clinical trials investigating antiangiogenic therapy in combination with immune checkpoint inhibitors in patients with mRCC.

**Clinicaltrials gov number/trial name (if applicable)**	**Phase**	**ICIs**	**AAs**	**Primary endpoint**	**Estimated study completion date**	**Therapy lines**
NCT02420821 (IMmotion151)	III	Atezolizumab	Bevacizumab	OS	2,021	First line
				PFS		
				PD		
NCT02853331 (KEYNOTE-426)	III	Pembrolizumab	Axitinib	PFS	2,022	First line
				OS		
NCT02684006 (JAVELIN Renal 101)	III	Avelumab	Axitinib	OS (PD-L1^+^)	2,024	First line
				PFS (PD-L1^+^)		
NCT02811861 (CLEAR)	III	Pembrolizumab	Lenvatinib	PFS	2,022	First line
NCT03937219 (COSMIC-313)	III	Nivolumab ipilimumab	Cabozantinib	PFS	2,024	First line
NCT03141177 (CheckMate 9ER)	III	Nivolumab	Cabozantinib	PFS	2,024	First line
NCT03172754	II	Nivolumab	Axitinib	ORR	2,024	First line
				AEs		
NCT03736330	II	Pembrolizumab	Axitinib	ORR	2,021	First line
NCT02501096	Ib/II	Pembrolizumab	Lenvatinib	MTD	2,020	Second line
				DLTs		
				ORR		
NCT03149822	I/II	Pembrolizumab	Cabozantinib	ORR	2,020	First line
NCT02210117	I	Ipilimumab	Bevacizumab	Safety	2,020	Neoadjuvant
				Tolerability		
NCT03680521	II	Nivolumab	Sitravatinib	ORR	2,020	First line

*AAs, Antiangiogenic Agents; ICIs, Immune Checkpoint Inhibitors; AEs, Adverse Events; DLTs, Dose-Limiting Toxicities; MTD, Maximum Tolerated Dose; ORR, Objective Response Rate; OS, Overall Survival; PD, Disease Progression; PD-L1^+^, PD-L1 positive patients; PFS, Progression-Free Survival*.

## Summary and Future Directions

The standard of mRCC management has experienced many radical changes over the past decade, and is in a state of revisions at present. Although the strategy of combining ICIs with AAs has a strong biological rationale, there is a lack of comparative studies juxtaposing novel combination front-line options, most of the available studies utilizing sunitinib monotherapy as a comparator arm. Taking this into account, the selection of first-line management for patients with mRCC should involve a thorough discussion and a comprehensive comparison of both efficacy and safety of available options.

In addition, rational and prospective trial designs with more extended follow-up period are necessary to figure out the remaining questions related to ICIs plus AAs approaches in mRCC care. Taken together, clinicians will need to confirm whether benefits of these agents are additive or synergistic and whether similar results can be achievable by sequentially using these agents. Clinicians also need to confirm whether the combination approach is always preferred or under what kind of circumstances can monotherapy be better.

Finally, as systemic therapies continue to evolve, it will be paramount to integrate multimodal approaches, including cytoreductive nephrectomy, stereotactic ablative radiotherapy and chimeric antigen receptor T cell (CAR-T) Immunotherapy, into the current treatment paradigm. For example, it is challenging to maintain peripheral CAR-T cell persistent. Early evidence in hematological malignancy models showed that PD-1 or PD-L1 might enhance CAR-T persistence (80). Local radiotherapy has also been observed to improve the response to ICI and AA in RCC (81, 82). In contrast to the shortage of treatment approaches and overwhelming toxicities before, treating mRCC is undoubtedly becoming increasingly complex with multiple trials performed and ongoing, and clinicians are faced with daunting challenges of staying abreast of the newest developments and integrating them into the care algorithms.

## Author Contributions

XZ, WH, and HZ: conception and design. CY: administrative support. XZ, WH, LG, and CY: provision of study materials or patients. XZ, WH, LG, and LS: collection and assembly of data. XZ and WH: manuscript writing. All authors: final approval of manuscript.

## Conflict of Interest

The authors declare that the research was conducted in the absence of any commercial or financial relationships that could be construed as a potential conflict of interest.

## Abbreviations

AA: Antiangiogenic Agent
CAR-T: Chimeric Antigen Receptor T-Cell
CTLA-4: Cytotoxic T-lymphocyte-Associated Protein 4
DC: Dendritic Cell
ICI: Immune Checkpoint Inhibitors
IMDC: International Metastatic Database Consortium
MDSC: Myeloid-Derived Suppressor Cell
MHC: Major Histocompatibility Complex
ORR: Objective Response Rate
OS: Overall Survival
PD-1/PD-L1: Programmed Death-1/Programmed Death Ligand-1
PFS: Progression-Free Survival
RCC: Renal Cell Carcinoma
TIL: Tumor Infiltrating Lymphocyte
TKI: Tyrosine Kinase Inhibitors
Treg: Regulatory T cell
VEGF: vascular endothelial growth factor.
